# *Streptococcus xiaochunlingii* sp. nov. E24 Isolated From the Oropharynx of Healthy Chinese Children

**DOI:** 10.3389/fmicb.2020.563213

**Published:** 2020-09-29

**Authors:** Yang Zou, Ye Sun, He Qi, Defeng Liu, Han Tian, Nan Wang, Xinming Li

**Affiliations:** Key Laboratory of Environmental Pollution and Microecology of Liaoning Province, Shenyang Medical College, Shenyang, China

**Keywords:** *Streptococcus*, phenotypic analyses, 16S rRNA, novel species, genome sequencing

## Abstract

A Gram-positive, α-hemolytic, catalase-negative, facultative anaerobic and non-motile coccus was isolated form throat swabs taken from the oropharynx of healthy children. The genome was shown to be 1.950,659 bp long and contained 42.03 mol% G + C bases with 1,942 protein-coding and 53 RNA coding genes. The results of 16S rRNA gene sequencing strongly suggested that the strain is a member of the *Streptococcus* genus, with 98.04, 98.11, and 97.34% similarities to *Streptococcus australis* ATCC 700641^T^, *Streptococcus rubneri* LMG 27207^T^ and *Streptococcus parasanguinis* ATCC 15912^T^, respectively. A sodA gene comparison exhibited a sequence identity of 92.6% with the closest strain *Streptococcus australis* ATCC 700641^T^. *In silico* DNA-DNA hybridization showed a highest DNA similarity value of 52% with *Streptococcus australis* ATCC 700641^T^. Comparing 18 biochemical traits, the similarities of the *Streptococcus* strain E24 were 72% with *Streptococcus rubneri* LMG 27207^T^, 78% with *Streptococcus australis* ATCC 700641^T^ and 44% with *Streptococcus parasanguinis* ATCC 15912^T^. We suggest that based on the genotypic and phenotypic results that the strain E24 is a novel species of the *Streptococcus* genus and propose the name *Streptococcus xiaochunlingii* sp. nov. E24.

## Introduction

The microbiota of the upper respiratory tract has been denoted as gatekeepers to respiratory health, since it prevent pathogens from overgrowing and disseminating toward the lungs (Man et al., 2017; Tsang et al., 2019). In the first week after birth, the upper respiratory microbiota of infants develop from a mixed toward a *Streptococcus viridans*-predominated bacterial community, followed by niche differentiation to *Staphylococcus aureus* and other bacteriae frequently leading to a composition of *Moraxella* spp., *Dolosigranulum* spp., and *Streptococcus* spp. (Bosch et al., 2016). In a previous study, we isolated *Streptococcus* strain E24 as one of 4 antagonistic strains which had bacteriostatic effects on *Klebsiella pneumoniae*, *Proteus vulgaris*, *Enterobacter cloacae*, and *Acinetobacter Baumannii* (Li et al., 2019).

In recent decades, due to improved phenotypic and molecular identification methods and with the development of the new taxonogenomics concept (Fournier et al., 2015), the genus *Streptococcus* has been significantly expanded with the addition of many newly discovered species (Huch et al., 2013; Ricaboni et al., 2017); more than 110 species are now recognized within the genus *Streptococcus*^1^. With 16S rRNA gene sequence analyses revealed that *Streptococcus* species were clustered into six groups comprising *S. anginosus*, *S. mitis*, *S. mutans*, *S. bovis*, *S. pyogenes*, and *S. salivarius* (Kawamura et al., 1995). Here we present the results of taxonogenomics concept analyses of a novel strain termed *Streptococcus* strain E24.

## Materials and Methods

### Collection of Bacterial Samples

Bacterial colonies from throat swabs of healthy children were cultivated aerobically for 24 h at 37°C on agar plates enriched with sheep blood (5%) (Becton Dickinson, Heidelberg, Germany). During streptococci isolation a single, circular, unpigmented and α-hemolytic colony of 0.5–1.0 mm diameter emerged, which was purified by repeated streaking and finally stored at −80°C. The study was conducted in accordance with the “Declaration of Helsinki” guidelines and approved by the Ethics Committee of Shenyang Medical College (approval number: No. 2015052902). Written informed consent was obtained from the legal representatives of the participating children.

### 16S rRNA sodA Gene Analyses

The nucleic acids of the strain were extracted for gene amplification and cloning of the 16S rRNA gene was accomplished by using a commercial DNA Purification Kit (Wizard Genomic DNA Purification Kit, Promega, Madison, WI, United States) and the 16S rRNA universal primers 27F/1492R (5′-AGAGTTTGATCMTGGCTCAG-3′ and 5′-GGYTACCTTGTTACGACTT-3′, respectively). The sequence obtained by sequencing was aligned using Basic Local Alignment Search Tool (BLAST) of NCBI. We collected 16S rRNA gene sequences of all known *Streptococcus* species from the NCBI database (Pontigo et al., 2015). Phylogenetic trees were constructed with two different algorithms, namely neighbor-joining (Saitou and Nei, 1987) and maximum-likelihood (Guindon and Gascuel, 2003) by close-neighbor-interchange (CNI) (search level = 2, random additions = 100) using MEGA version 7.0 (Kumar et al., 2016) and the reliability of the nodes was estimated by bootstrap analysis (1000 replications) (Felsenstein, 1985). Additionally, we analyzed the sodA gene since it has been proposed for phylogenetic characterization especially for differentiating streptococci (Poyart et al., 1998) with the primers forward: 5′-CCITAYICITAYGAYGCIYTIGARCC-3′ and reverse: 5′-ARRTARTAIGCRTGYTCCCAIACRTC-3′ leading to a 430 bp fragment.

### Biochemical and Phenotypic Characterizations

Isolate growths were examined in aerobic, anaerobic and microaerophilic atmospheres, with or without 5% CO_2_ and at 4, 15, 22, 30, 35, 37 and 42°C using nutrient agar containing sheep blood (5%, Solarbio, Beijing, China) (Facklam and Elliott, 1995). The nutrient agar consisted of peptone 10.0 g/L, beef powder 3.0 g/L sodium chloride 5.0 g/L, agar 15.0 g/L with pH of 7.3 ± 0.1 (Qingdao Hope Bio-Technology Co., Ltd., Qingdao, China). Cell motility and chain building was evaluated with the aid of a Leica DM1000 light microscope (Leica Microsystems, France). Specimens were also examined after the classical Gram straining (Austrian, 1960). The susceptibility of each isolates to salt was tested by growing the strains in the presence of 2.5, 3.5, 4.5 or 6.5 g/L NaCl. Moreover, nine different pHs were tested: 4.0, 5.0, 5.5, 6.0, 6.5, 7.0, 7.5, 8.0, and 9.0 (Supplementary Table 1).

Cell sizes were calculated from images captured on a scanning electron microscope. Bacteria were fixed in glutaraldehyde phosphate (2.5%) buffer overnight at 4°C. They were then washed twice with phosphate buffer (PBS), fixed for 30 min in a 1% osmium tetroxide solution (SPI supplies Inc., West Chester, PA, United States) and then washed 3 times with PBS before being dehydrated with 50, 70, 80, 90, 95 (twice), and 100% ethanol (three times), respectively (5 min per ethanol concentration). After natural drying, each sample was subjected to ion sputtering and then the gold was sprayed onto the surface and then viewed on a Hitachi 3400N scanning electron microscope (Hitachi High Technologies, Japan).

Sporulation was tested with *Streptococcus* strain E24 solutions (concentration: 1 × 10^6^ cfu/mL) divided into two groups, from which one group was heated to 80–90°C for 20 min and the other served as control. Then 50 μl solutions of each group were applied on nutrient agar plates containing 5% sheep blood. The strains were incubated under normal conditions at 37°C overnight to observe the bacterial colony formation. No colony growth was observed on the plate of the heat treatment group on the next day.

### Biochemical Assays

Isolates were characterized with Rapid ID32 Strep, API ZYM and API 50CH systems (bioMérieux, Marcy-l’Étoile, France) as well as catalase (Becton Dickinson, Franklin Lakes, NJ, United States) and oxidase (Qingdao Hope Bio-Technology Co., Ltd., Qingdao, China) assays were carried out separately.

### Susceptibility to Antibiotics

The antibiotic susceptibility of the strain *Streptococcus* strain E24 was determined using disk diffusion (Le Page et al., 2015) on Mueller-Hinton E agar (bioMérieux, Marcy-l’Étoile, France) consisting of casamino acid 17.5 g/L, beef extract 2.0 g/L, soluble starch 1.5 g/L Agar 17.0 g/L at pH 7.2 ± 0.2 (25°C) (Qingdao Hope Bio-Technology Co., Ltd., Qingdao, China). The antimicrobial agents tested were: vancomycin, ceftriaxone, cefepime, cefotaxime, Rina Thiazole Amine and chloramphenicol (all at 30 μg); ampicillin and penicillin (10 μg); and clindamycin (2 μg).

### Analysis of Fatty Acids in Bacteria

Fatty acid methyl esters (FAMEs) were analyzed using a gas chromatography/mass spectrometry [see (Dione et al., 2016) and (Sasser, 1990)]. FAMEs were prepared from a sample consisting of 40 mg of bacterial biomass, separated using an MS column (Elite 5) and examined with a mass spectrometry (Clarus 500-SQ 8 S, PerkinElmer, France) followed by appropriate searches of spectral databases.

### Extraction of DNA and Sequencing of the Genome

Genomic DNA was extracted using the SDS methodology (Lim et al., 2016). Agarose gel electrophoresis (HE-120, Tanon Science & Technology Inc., Shanghai, China) was used to detect the DNA, which was analyzed and quantified with a Qubit 2.0 Fluorometer (Life Technologies, Carlsbad, CA, United States). 1 μg samples were used as preparations of DNA and sequence libraries were constructed using a NEBNext Ultra DNA Library Prep Kit for Illumina (New England Biolabs Inc., Ipswich, MA, United States), with appropriate index codes. Samples were sonicated to sizes of about 350 bp and fragments end-polished, A-tailed and ligated for subsequent amplification by PCR. The PCR products were purified and libraries evaluated to determine the distribution of sizes using real-time PCR (Agilent 2100 Bioanalyzer, Agilent Technologies, Palo Alto, CA, United States). Beijing Novogene Bioinformatics Technology Co., Ltd sequenced the genome (Illumina NovaSeq PE150, Illumina Inc., San Diego, United States).

### Genome Sequencing Assembly and Annotation

After preprocessing, appropriate data were gathered using SOAP (version 2.04) (Li et al., 2008, 2010), SPAdes (Bankevich et al., 2012) and ABySS (Simpson et al., 2009) assembly software, and finally integrated using CISA software. The preliminary assembly results were optimized and holes filled with GapCloser (version 1.12) to obtain the final assembly result. Fragments below 500 bp were filtered out. Encoded genes were predicted using GeneMarkS software. The protein-coding sequences produced with BLAST were annotated by comparison with the NR, GO, KEGG, COG, PFAM, TCDB, and Swiss-Prot protein databases.

### Genome Comparison

Average nucleotide identity (ANI) was used to assess genomic similarity and to define species with values of 95%. The ANI analysis was performed with OAT (Yoon et al., 2017) and DNA-DNA hybridization (DDH) was used to assess the genomic similarity and to define species with 70% hybridization values (Tindall et al., 2010). The Genome-to-Genome Distance Calculator (GGDC) (Meier-Kolthoff et al., 2013) was employed to conduct a digital DNA-DNA hybridization (dDDH) analysis. The adjacent to the *Streptococcus* strain E24 located strains were selected based on BLAST alignment and the phylogenetic tree constructed from 16S rRNA gene results data.

## Results

### Phylogenetic Analysis

**FIGURE 1:**
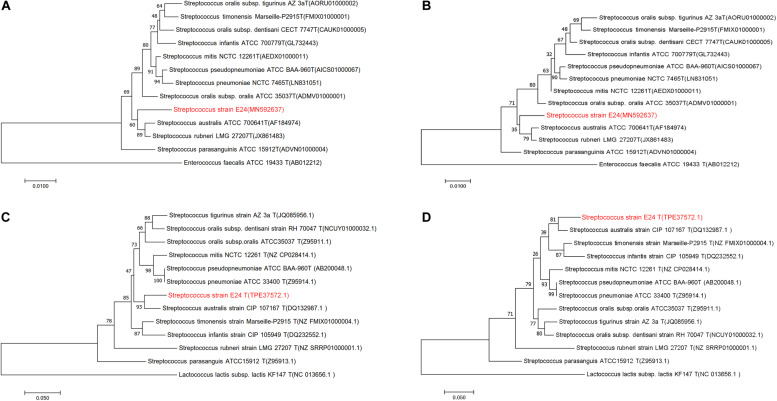
Phylogenetic trees of *Streptococcus* strain E24 and closely related members of the *Streptococcal* species groups. **(A)** Neighbor-joining and **(B)** maximum-likelihood 16S rRNA gene sequences comparisons, **(C)** neighbor-joining and **(D)** maximum-likelihood sodA gene sequences comparisons. Sequences of *Enterococcus faecalis* and *Lactococcus lactis* subsp. *lactis* were used as out-groups. Bootstrap values ≥ 50 are indicated and the bars indicate 0.01 and 0.05 substitutions per site.

The newly determined 16S rRNA (Figures 1A,B) isolate gene sequence was aligned with sequences of other *Streptococcus* species retrieved from GenBank. Strain *Streptococcus* strain E24 shared 98.04, 98.11, and 97.34 % 16S rRNA gene sequence similarity with *S. australis* ATCC 700641^T^, *S. rubneri* LMG 27207^T^ and *S. parasanguinis* ATCC 15912^T^, respectively. The housekeeping gene sodA (Figures 1C,D) of *Streptococcus* strain E24 shared 94.94, 92.74, and 90.80% similarities with *S. australis* ATCC 700641^T^, *S. timonensis Marseille*-P2915^T^ and *S. infantis* ATCC 700779^T^, respectively. Figures 1A,C represent phylogenetic trees constructed with the neighbor-joining and Figures 1B,D phylogenetic trees constructed with the maximum-likelihood method. Taken together, the phylogenetic analyses demonstrated that *Streptococcus* strain E24 represents a novel species within the genus *Streptococcus*. The 16S rRNA gene sequences of *Streptococcus* strain E24 is recorded in the NCBI database (accession no. MN592637).

### Phenotypic Characteristics

**FIGURE 2:**
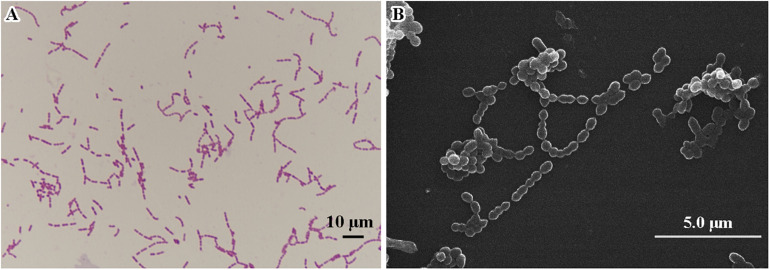
Microscopic imaging of *Streptococcus* strain E24 **(A)** Gram stained coccus under light microscope 10 × 100 magnification, scale bar = 10 μm. **(B)** Transmission electron microscope image (operating voltage of 30.0 kV), scale bar = 5.0 μm.

Morphological features were determined using cells cultured aerobically at 37°C on enriched nutrition agar (*vide supra*) for 24 h, when the bacteria became punctate, gray and formed α-hemolytic colonies having edges that were undulated. Gram staining was performed using the classical Gram stain procedure (Austrian, 1960) and light microscopy images showed Gram-positive cocci (Figure 2A). Cells had an average diameter of about 5 μm as viewed by an electron microscopy (Figure 2B). Salt tolerance was determined by the growth of the strains in the presence of 2.5 g/L. The isolate was found to be unable to grow at or below 22°C but could grow at up to 42°C, with 37°C being the optimal temperature. The cells were facultative anaerobe, and the pH for optimal growth was 7 (Table 1 and Supplementary Table 1).

**TABLE 1:** Classification and general features of *Streptococcus* strain E24.

Property	Result
α-Hemolytic	+
Gram stain	Positive
Cell shape	Cocci
Motility	Non-motile
Sporulation	Non-spore forming
Temperature range	22–42°C
Optimum temperature	37°C
Habitat	Child respiratory tract
Oxygen requirement	Facultative aerobic
Salinity	0–2.5 g/L
Optimum salinity	0 g/L
pH optimum	7

*Streptococcus strain E24 has been deposited in the Korean Agricultural Culture Collection (deposition no. KACC 21425) and Guangdong Microbial Culture Collection Center (deposition no. GDMCC 1.1634). The genome sequence and 16S rRNA gene sequence were recorded in the NCBI database (accession no. VFSG00000000; accession no. MN592637).*

### Biochemical Characterization and FAME Profile

Catalase and oxidase tested negative and *Streptococcus* strain E24 did not hydrolyse arginine, aesculin or starch except hippurate and was negative for esterase, leucin arylamidase, valin arylamidase and β-D-galactosidase, but β-D-glucuronidase, β-D-glucosidase and alkaline phosphatase produced positive results. *Streptococcus* strain E24 does not produce acid from glycerol, erythritol, d- or l-arabinose, d-ribose, d- or l-xylose, D-adonitol, methyl β-D-xyloside, d-fructose-l-sorbose, l-rhamnose, dulcitol, d-mannitol, methyl α-D-mannopyranoside, methyl α-D-glucopyranoside, amygdalin, arbutin, aesculin, salicin, cellobiose, d-maltose, trehalose, inulin, melezitose, starch, glycogen, xylitol, gentiobiose, d-raffinose, d-melibiose, d-sorbitol, d-lyxose, d-tagatose, d- or l-fucose, d- or l-arabitol or 2- or 5-ketogluconate. Utilization of d-lactose is variable. *Streptococcus* strain E24 was capable of producing acid from D-galactose, D-glucose, D-mannose, inositol, N-acetylglucosamine and sucrose. Different biochemical characteristics between the novel *Streptococcus* strain E24 (strain number 1) and other members of the *Streptococcus* genus are shown in Table 2. Hexadecanoic acid (16:00), (9Z)-9-octadecenoic acid (18:1 w9c), octadecanoic acid (18:00) and (11Z)-11-octadecenoic acid (18:1 w7c) comprised 62% of all fatty acids (Table 3).

**TABLE 2:** Differential characteristics of *Streptococcus* strain E24 in comparison with other related organisms.

Strains	1	2	3	4	5	6	7	8	9
Hemolysis	α	β	α	α	α	α	α	α	α
Acetoin (Voges Proskauer)	-	-	-	-	-	-	-	-	-
**Hydrolysis of**:									
Arginine	-	-	+	+	-	-	-	v	+
Aesculin	-	-	-	+	v	-	-	-	v
Hippurate	+	-	-	-	-	-	-	-	-
Starch	-	-	-	+	v	-	-	-	v
**Production of**:									
α-D-galactosidase	+	-	-	+	-	-	-	+	-
β-D-galactosidase	-	-	-	+	+	+	-	+	+
β-D-glucosidase	+	-	-	-	-	-	v	-	-
**Acid from**:									
D-lactose	v	+	+	+	+	+	+	+	+
D-mannitol	-	-	-	-	+	-	v	-	-
D-melibiose	-	-	-	+	v	-	-	v	-
D-raffinose	-	+	-	+	v	-	-	v	+
D-ribose	-	-	-	-	v	-	-	v	-
D-sorbitol	-	-	-	-	-	-	-	-	-
D-sucrose	+	+	+	+	+	+	v	+	+
D-tagatose	-	-	-	+	-	-	-	-	-
D-trehalose	-	-	-	+	-	-	-	-	-

*Strains: 1, Streptococcus strain E24; 2, S. rubneri (Handley et al., 1991; Willcox et al., 2001; Huch et al., 2013); 3, S. australis (Willcox et al., 2001); 4, S. parasanguinis (Whiley et al., 1990; Willcox et al., 2001); 5, S. oralis (Bridge and Sneath, 1982; Whiley and Hardie, 2009); 6, S. infantis (Kawamura et al., 1998; Whiley and Hardie, 2009); 7, S. pseudopneumoniae (Arbique et al., 2004; Zbinden et al., 2012);8, S. mitis (Whiley and Hardie, 2009); and 9, S. pneumoniae (Whiley and Hardie, 2009). +, positive; -, negative; v, variable.*

**TABLE 3:** Fatty acid profiles of *Streptococcus* strain E24.

Fatty acid	Percent (%)
16:00	26.03
18:1 w9c	13.47
18:00	11.88
Summed feature 8 (18:1 w7c)	11.01
Summed feature 5 (18:2 w6,9c/18:0 ante)	7.9
14:00	7.32
Summed feature 3 (16:1 w7c/16:1 w6c)	6.82
16:1 w9c	6.46
12:00	2.15
16:1 w5c	1.03
17:00	0.91
17:1 iso w5c	0.76
20:4 w6,9,12,15c	0.75
15:00	0.73
17:1 w8c	0.56
17:1 anteiso w9c	0.51
19:0 iso	0.5
17:0 iso	0.46
17:0 anteiso	0.46
13:0 2OH	0.29

### Antibiotic Susceptibility

Antibiotic susceptibility tests of *Streptococcus* strain E24 revealed susceptibility to vancomycin, cefepime, Rina thiazole amine, penicillin, chloramphenicol and clindamycin, but resistance to ceftriaxone, ampicillin, and cefotaxime.

### Genome Properties

**FIGURE 3:**
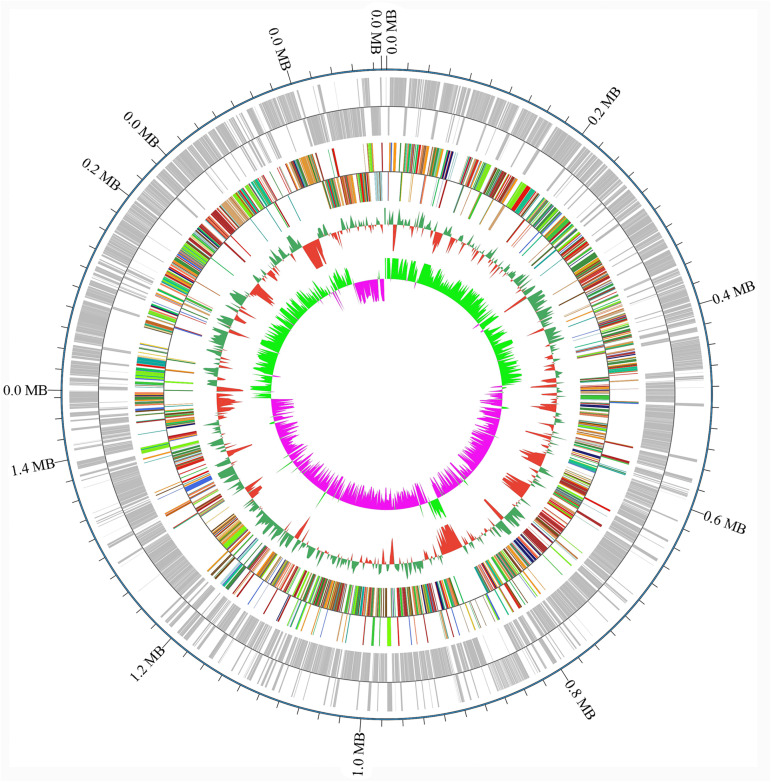
Levels of gene counts and nucleotide content of the *Streptococcus* strain E24 genome.

A draft of the genome of *Streptococcus* strain E24 revealed that it was comprised of 1,950,659 bp with a 42.03% G + C content (Figure 3). It is comprised of five scaffolds (five contigs). Among the 1,994 genes predicted, 1,942 encoded proteins and 52 RNAs (3 genes were 5S rRNA, 1 gene 16S rRNA, 1 gene 23S rRNA and 47 genes tRNA). Of these, 1,525 genes (78.52%) were specified with putative functions according to COG or NR BLAST analysis. Twenty-two genes were ORFs (1.13%) and the remaining genes were responsible for hypothetical proteins. Table 4 shows the COG functional categories of the sorted genes. The sequence of the genome was recorded in the NCBI database (accession no. VFSG00000000).

**TABLE 4:** Number of genes associated with 26 general COGs functional categories.

Code	Value	% of total^a^	Description
A	0	0	RNA processing and modification
B	0	0	Chromatin structure and dynamics
C	49	3.21	Energy production and conversion
D	24	1.57	Cell cycle control, cell division, chromosome partitioning
E	144	9.44	Amino acid transport and metabolism
F	71	4.66	Nucleotide transport and metabolism
G	128	8.39	Carbohydrate transport and metabolism
H	65	4.26	Coenzyme transport and metabolism
I	44	2.89	Lipid transport and metabolism
J	190	1.25	Translation, ribosomal structure and biogenesis
K	109	7.15	Transcription
L	85	5.57	Replication, recombination and repair
M	103	6.75	Cell wall/membrane/envelope biogenesis
N	9	0.59	Cell motility
O	64	4.20	Posttranslational modification, protein turnover, chaperones
P	64	4.20	Inorganic ion transport and metabolism
Q	19	1.25	Secondary metabolites biosynthesis, transport and catabolism
R	117	7.67	General function prediction only
S	91	5.97	Function unknown
T	67	4.39	Signal transduction mechanisms
U	18	1.18	Intracellular trafficking, secretion, and vesicular transport
V	49	3.21	Defense mechanisms
W	2	0.13	Extracellular structures
X	13	0.85	Mobilome: prophages, transposons
Y	0	0	Nuclear structure
Z	0	0	Cytoskeleton

*COGs, clusters of orthologous groups.^a^Total based on the number of protein-coding genes found in the genome.*

### Comparisons of Genomes

The genome sequence length of *Streptococcus* strain E24 (1,950 Mb) was less than *S. pseudopneumoniae* ATCC BAA-960^T^ (2,086 Mb), *S. tigurinus* AZ 3a^T^ (2,185 Mb), *S. pneumoniae* NCTC 7465^T^ (2,161 Mb), *S. parasanguinis* ATCC 15912^T^ (2,154 Mb) and *S. sanguinis* ATCC 10556^T^ (2,303 Mb), but greater than that of *S. dentisani* CECT 7747^T^ (1,884 Mb), *S. infantis* ATCC 700779^T^ (1,857 Mb), *S. mitis* NCTC 12261^T^ (1,916 Mb) and *S. oralis* ATCC 35037^T^ (1,914 Mb). The G + C contents of *Streptococcus* strain E24 (42.03%) was greater than that of *S. pseudopneumoniae* ATCC BAA-960^T^ (39.8%), *S. dentisani* CECT 7747^T^ (41.1%), *S. infantis* ATCC 700779^T^ (38.9%), *S. mitis* NCTC 12261^T^ (41.1%), *S. oralis* (41.4%), *S. pneumoniae* NCTC 7465^T^ (39.7%), *S. parasanguinis* ATCC 15912^T^ (41.7%) and *S. tigurinus* AZ 3a^T^ (40.3%), but less than that of *S. sanguinis* ATCC 10556^T^ (43.2%).

The distribution of genes into categories of COG was identical for all genomes investigated. To determine similarities between genomes between strains, we assigned two parameters, namely dDDH, which had a high correlation with DDH (Auch et al., 2010), and AGIOS, which was independent of DDH. The degree of nucleotide sequence similarity and numbers of orthologous genes between different genomes was also examined. Concerning dDDH values, it was found that the range varied between 23.5 and 57.8% within the 11 comparison strains, and 24 to 52% when *Streptococcus* strain E24 was matched with 11 comparison strains (Table 5).

**TABLE 5:** Pairwise comparison of *Streptococcus* strain E24 with nine other *Streptococcal* species using genome-to-genome distances and formula 2 (DDH estimates based on identities/high-scoring segment pair length).

	1	2	3	4	5	6	7	8	9	10	11	12
1	100%	52%	37.5%	26.3%	24.20%	29%	27.6%	24%	24.8%	25.8%	25%	25.5%
2		100%	40.3%	27.40%	24.00%	28.10%	28.70%	24.00%	24.80%	24.90%	25.60%	26.00%
3			100%	26.00%	24.70%	29.30%	27.40%	24.70%	24.60%	25.00%	25.6%	26.10%
4				100%	24.50%	26.00%	26.50%	23.50%	24.80%	24.60%	24.60%	25.60%
5					100%	25.20%	25.70%	44.00%	48.60%	31.60%	32.10%	31.80%
6						100%	37.40%	25.50%	25.50%	26.20%	26.60%	25.80%
7							100%	25.40%	26.30%	26.70%	26.30%	25.70%
8								100%	44.90%	31.30%	31.30%	31.10%
9									100%	32.30%	31.70%	31.80%
10										100%	48.20%	57.80%
11											100%	47.20%
12												100%

*Strains: 1. Streptococcus strain E24; 2. S. australis (Willcox et al., 2001); 3. S. rubneri (Handley et al., 1991; Willcox et al., 2001; Huch et al., 2013); 4. S. parasanguinis (Whiley et al., 1990; Willcox et al., 2001); 5. S. oralis subsp. Tigurinus (Zbinden et al., 2012); 6. S. timonensis (Ricaboni et al., 2017); 7. S. infantis (Kawamura et al., 1998; Whiley and Hardie, 2009); 8. S. oralis subsp. Dentisani (Jensen et al., 2016); 9. S. oralis subsp. Oralis (Jensen et al., 2016); 10. S. pseudopneumoniae (Arbique et al., 2004; Zbinden et al., 2012); 11. S. mitis (Whiley and Hardie, 2009); 12. S. pneumoniae (Whiley and Hardie, 2009).*

## Discussion

The phenotypic analysis obtained from API strips (Table 2) revealed, that the *Streptococcus* strain E24 was the only one which hydrolyzed hippurate and there were essential differences between the nine analyzed streptococci, which has been described also for other *Streptococcus* strains in which the probability that a strain was correctly identified by nine phenotypic tests was 90–100% (Raemy et al., 2013). The similarities of the 16S rRNA gene sequences from *Streptococcus* strain E24 with the closest related streptococci were 98.04, 98.11, and 97.34% for *Streptococcus australis* ATCC 700641^T^, *Streptococcus rubneri* LMG 27207^T^ and *Streptococcus parasanguinis* ATCC 15912^T^, whereas the sodA gene comparison exhibited a sequence identity of 92.6% with the closest strain *Streptococcus australis* ATCC 700641^T^. These similarities are in line with another newly identified *Streptococcus* strain exhibiting 98.7 and 92.6% similarities of 16S rRNA and rpoB gene sequences between the *Streptococcus timonensis* sp. nov. and the *Streptococcus infantis* strain JCM 10157^T^ (Ricaboni et al., 2017). There were also essential differences of G + C contents and genome sequence lengths between *Streptococcus* strain E24 and eight other streptococci, but also within the 8 streptococci species, which has been described also for other *Streptococcus* strains (Thompson et al., 2013) and attributed to frequent occurrence of horizontal gene transfers (Bellanger et al., 2009; Harvey et al., 2011; Zhang et al., 2011). Most obviously, the dDDH analysis revealed 24 to 52% similarities between *Streptococcus* strain E24 and 11 comparison strains and the similarities between the 12 different streptococci strains was between 23.5 and 57.8%, which is less than the proposed limit of 70% similarity for discrimination of *Streptococcus* species (Auch et al., 2010; Thompson et al., 2013).

In summary, based on the genotypic, phylogenetic and phenotypic results, it is concluded that the *Streptococcus* strain E24 is a novel species of the genus *Streptococcus*, which is distinct from its closest phylogenetic neighbors *Streptococcus australis* ATCC 700641^T^, *Streptococcus rubneri* LMG 27207^T^ and *Streptococcus parasanguinis* ATCC 15912^T^ and for which the name *Streptococcus xiaochunlingii* sp. nov.E24 is proposed.

## Description of *Streptococcus xiaochunlingii* sp. nov. E24

*Streptococcus xiaochunlingii* (xiao’chun’ lingi.i. N.L. gen masc. named after Professor Xiao Chunling, who is the leader of the laboratory in which the strain has been isolated).

*Streptococcus* strain E24 is a non-motile, non-spore-forming, facultative anaerobic and Gram-positive coccus, isolated from the oropharynx of healthy children. Growth is achieved under aerobic, microaerophilic and anaerobic atmospheres and had a temperature growth range of 22 to 42°C, with the optimum temperature being 37°C. After 48 h of aerobic incubation on 5% sheep’s blood–enriched nutrition agar, colonies are pinpoint, grayish and α-hemolytic, with undulated edges and with a diameter of 0.5 to 1 mm. Cells are roughly round with a 0.5 μm diameter. Cells did not hydrolyse arginine, aesculin or starch except hippurate and were negative in tests for esterase, leucin arylamidase, valin arylamidase and β-D-galactosidase. Positive for β-D-glucuronidase, β-D-glucosidase and alkaline phosphatase. Does not produce acid from glycerol, erythritol, d- or l-arabinose, d-ribose, d- or l-xylose, d-adonitol, methyl β-D-xyloside, d-fructose-l-sorbose, l-rhamnose, dulcitol, d-mannitol, methyl α-D-mannopyranoside, methyl α-D-glucopyranoside, amygdalin, arbutin, aesculin, salicin, cellobiose, d-maltose, trehalose, inulin, melezitose, starch, glycogen, xylitol, gentiobiose, d-lyxose, d-raffinose, d-tagatose, d- or l-fucose, d- or l-arabitol, d-sorbitol, d-melibiose or 2- or 5-ketogluconate. Utilization of d-lactose is variable. They were capable of producing acid from D-galactose, D-glucose, d-mannose, inositol, N-acetylglucosamine and sucrose. Catalase and oxidase tested negative. The genome was shown to be 1.950,659 bp long and contained 42.03 mol% G + C bases with 1,942 protein-coding and 53 RNA coding genes. The results of 16S rRNA gene sequencing strongly suggested that the strain was a member of the *Streptococcus* genus, with 98.04, 98.11, and 97.34% similarities to *Streptococcus australis* ATCC 700641^T^, *Streptococcus* rubneri LMG 27207^T^ and *Streptococcus parasanguinis* ATCC 15912^T^, respectively. A sodA gene comparison exhibited a sequence identity of 92.6% with the closest strain *Streptococcus australis* ATCC 700641^T^. *In silico* DNA–DNA hybridization showed a closest DNA similarity value of 52% with *Streptococcus australis* ATCC 700641^T^. *Streptococcus* strain E24 has been deposited in the Korean Agricultural Culture Collection (deposition no. KACC 21425) and Guangdong Microbial Culture Collection Center (deposition no. GDMCC 1.1634). The genome and 16S rRNA gene sequences are recorded in the NCBI database (accession no. VFSG00000000; accession no. MN592637).

## Data Availability Statement

The datasets presented in this study can be found in online repositories. The names of the repository/repositories and accession numbers can be found below: https://www.ncbi.nlm.nih.gov/, VFSG00000000; https://www.ncbi.nlm.nih.gov/, MN592637.

## Ethics Statement

The studies involving human participants were reviewed and approved by Ethics Committee of Shenyang Medical College (approval number: No. 2015052902). Written informed consent to participate in this study was provided by the participants’ legal guardian/next of kin.

## Author Contributions

XL and YZ were responsible for the conception and design of the study, drafted, commented, and revised the manuscript. XL, YZ, YS, HQ, and DL were in charge of statistical analysis. All authors were responsible for data acquisition and analysis, read and approved the final version of this manuscript.

## Conflict of Interest

The authors declare that the research was conducted in the absence of any commercial or financial relationships that could be construed as a potential conflict of interest.

## Footnotes

1
http://www.bacterio.net
