# Supplementary material for: Streptococcus xiaochunlingii sp. nov. E24 Isolated From the Oropharynx of Healthy Chinese Children
Source: Front Microbiol. 2020 Sep 29;11:563213. doi: 10.3389/fmicb.2020.563213 (PMC7550633; doi:10.3389/fmicb.2020.563213)
Supplement: Supplementary file 1 [file Table_1.DOCX]

**Supplementary Table 1.** The results of strain growth in different conditions

| **Atmosphere test (cultured at 37°C for 24 h)** | | | | | | | | | | | | | | | | |
| --- | --- | --- | --- | --- | --- | --- | --- | --- | --- | --- | --- | --- | --- | --- | --- | --- |
| Atmosphere | +5% CO2 | | | | | Normal aerobic | | | | | | Anaerobic bag culture | | | | |
| Number of bacterial colonies | +++ | | | | | +++ | | | | | | ++(+) | | | | |
| **Temperature test (cultured under aerobic condition for 24 h)** | | | | | | | | | | | | | | | | |
| Temperature (°C) | 4 | | 15 | | 22 | | 30 | | 35 | | 37 | | 42 | | 45 | |
| Number of bacterial colonies | - | | - | | + | | + | | ++ | | +++ | | ++ | | - | |
| **Salt tolerance test (cultured under aerobic condition at 37°C for 24 h)** | | | | | | | | | | | | | | | | |
| NaCl concentration (g/L) | 2.5 | | | | 3.5 | | | | 4.5 | | | | 6.5 | | | |
| Number of bacterial colonies | +++ | | | | - | | | | - | | | | - | | | |
| **pH test (cultured under aerobic condition at 37°C for 24 h)** | | | | | | | | | | | | | | | | |
| pH | 4.0 | 5.0 | | 5.5 | | 6.0 | | 6.5 | | 7.0 | | 7.5 | | 8.0 | | 9.0 |
| Number of bacterial colonies | - | - | | + | | + | | ++ | | +++ | | + | | + | | - |

Note: Every 50 ul strain solution (concentration: 1*10^6^cfu/ml) was used to inoculate with each plate.

There's no a “+” difference between growth of strains with or without carbon dioxide.

-: no growth

+: < 100 (colony)

++: 100 – 500 (colony)

+++: > 500 (colony)
